# Extraction of phenolics and anthocyanins from purple eggplant peels by multi-frequency ultrasound: Effects of different extraction factors and optimization using uniform design

**DOI:** 10.1016/j.ultsonch.2022.106174

**Published:** 2022-09-23

**Authors:** Jianqing Liao, Hongkun Xue, Junling Li

**Affiliations:** a College of Physical Science and Engineering, Yichun University, 576 Xuefu Road, Yichun, Jiangxi 336000, China; b College of Traditional Chinese Medicine, Hebei University, No. 342 Yuhua East Road, Lianchi District, Baoding 071002, China; c College of Chemistry and Bioengineering, Yichun University, 576 Xuefu Road, Yichun, Jiangxi 336000, China

**Keywords:** Ultrasound extraction, Uniform design, Phenolic content, Monomeric anthocyanins, Parameter optimization

## Abstract

In this work, multi-frequency ultrasound (working modes for the single-, dual- and tri-frequency in simultaneous ways) was applied to extract bioactive compounds from purple eggplant peels. The single-factor experiments were performed by varying six independent variables. A six-level-five-factor uniform design (UD) was further employed to evaluate the interaction effects between different factors. It was found that extraction temperature and extraction time significantly affected the total phenolic content (TPC), whereas the total monomeric anthocyanins (TMA) was mainly influenced by ethanol concentration, extraction temperature and solid-liquid ratio. Based on partial least-squares (PLS) regression analysis, the optimal conditions for TPC extraction were: 53.6 % ethanol concentration, 0.336 mm particle size, 44.5 °C extraction temperature, 35.2 min extraction time, 1:43 g/mL solid-liquid ratio, and similar optimal conditions were also obtained for TMA. Furthermore, the TPC and TMA extraction were investigated by ultrasound in different frequencies and power levels. Compared with single-frequency (40 kHz) and dual-frequency ultrasound (25 + 40 kHz), the extraction yield of TPC and TMA with tri-frequency ultrasound (25 + 40 + 70 kHz) increased by 23.65 % and 18.76 % respectively, which suggested the use of multi-frequency ultrasound, especially tri-frequency ultrasound, is an efficient strategy to improve the TPC and TMA extracts in purple eggplant peels.

## Highlights

• Multi-frequency ultrasound was firstly applied to extract TPC and TMA from eggplant.
• UD Combined with PLS was firstly used to determine the optimal extraction conditions.
• Impact of ultrasound in different frequencies and powers on TPC and TMA were studied.
• The sound extraction efficiency of TFU was superior to non-ultrasound, SFU and DFU.
• The application prospect of multi-frequency ultrasound in extraction field is great.

## Graphical abstract.

**Figure:**
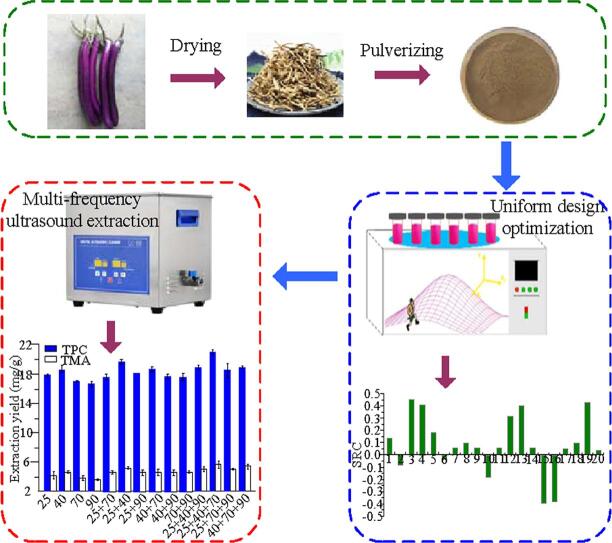

## 1 Introduction

Eggplant (*Solanum melongena* L.), one of the popular species like tomato, pepper and potato, is widely distributed in South-east Asia such as China, Thailand and India [1], [2]. It was reported that about 55 million tons of eggplant were produced worldwide in 2019, and more than 80 % of this production was distributed between China and India [3]. Nowadays, a large number of eggplant varieties ranging in size (from a few grams to more than a kilo), shape (semi-long, long, oblong, globular, and ovoid), and color (purple, white, violet, and green) are cultivated in the temper, subtropics and atetropics areas [4], [5].

Eggplant fruit is important items of the human diet due to its many cooking ways and various used states such as fresh, dried and preserved. It was reported that eggplant is a good source of minerals (magnesium, potassium), vitamins (vitamins B1, B6 and K), dietary fiber and phytochemicals, especially valuable phenolic compounds, which are extensively used to decrease the blood cholesterol rate in humans [6] and to treat various health disorders including diabetes bronchitis, arthritis and asthma [7], [8], [9]. Additionally, eggplant peels are rich in anthocyanins which is one of the most important flavonoids as well as the determination of the eggplant peel colour. The beneficial effects of eggplants are closely linked to the presence of its antioxidant properties that is ranked among other vegetables in terms of oxygen radical absorbance capacity [10]. Owing to the many benefits of phenolic compounds, there is interest in the extraction of phenolic compounds and anthocyanins from various plant materials to produce higher value-added products.

The extraction efficiency of plant active ingredients is greatly influenced by several factors including their storage conditions, chemical nature, especially extraction conditions as well as the use of extraction methods [11]. In many previous studies, the traditional extraction methods such as solvent extraction (decoction, digestion, maceration, hot continuous extraction), distillation, cold compression, countercurrent extraction, as well as various newer extraction techniques including pressurized liquid extraction, supercritical fluid extraction, microwaves assisted extraction and ultrasound assisted extraction were successfully applied to extract the vegetal compounds [12], [13], [14], [15], [16], [17]. Compared with the traditional extraction methods, extraction assisted by ultrasound has exhibited many advantages such as less amount of solvent, shorter extraction time and lower operating temperature with higher extraction rate. These benefits derived from the intense cavitation effect produced by ultrasound in liquid media [18], [19].

Ultrasound can be applied using two types of devices namely probe-type ultrasound and bath-type ultrasound. The probe-type ultrasound is high reproducibility and more powerful because the ultrasonic intensity can be transmitted through a smaller surface (only the tip of the probe). However, compared with the bath-type ultrasound, probe-type ultrasound is based on only one transducer which corresponds to only one frequency as the source of ultrasound power, while bath-type ultrasound device is the most commonly known type of ultrasonic equipment that consists of one or more ultrasonic transducers with more frequencies, which is beneficial to expand the application of multi-frequency ultrasound in the field of ultrasound extraction. Moreover, bath-type ultrasound device is usually equipped with temperature control, and it is readily cheap and lots of samples can be extracted simultaneously [20], [21].

As mentioned above, the ultrasonic frequency used in probe-type ultrasound device is single, which can easily produce several adverse standing waves for the extraction process, therefore to restrict the interaction between the target sample and acoustic wave. Compared with the single acoustic wave, the collaborative use of multiple sound waves produced by the ultrasound bath-type device can effectively reduce the influence of standing waves and improve the uniformity of ultrasonic field distribution, so that the sound energy can be effectively utilized. It is well known that ultrasonic frequency is one of the important variables affecting cavitation effect. There is a very important correlation between ultrasonic frequency and ultrasonic chemical effect. The generation and intensity of ultrasonic cavitation in liquid medium change with the change of ultrasonic frequency. For low frequency, the physical effect is stronger than the chemical effect due to the small number of cavitation bubbles, while for high frequency the cavitation effect needs to be generated with greater amplitude and intensity [22]. Therefore, the combination of multiple frequencies is expected to obtain higher extraction rate [23]. Cao et al. [24] reported that higher Pb (II) content was obtained from wheat samples by dual-frequency ultrasound extraction. Naa Yarley et al. [25] also found that the polysaccharides yield from crude sorghum leaf with multi-frequency ultrasound increased by 26.69 %. Moreover, a large number of similar results have been reported under the use of multi-frequency ultrasound in recent years [26], [27], [28], [29], [30], [31]. For the extraction of phenolic compounds from eggplant, although several studies have been performed by ultrasound [32], [33], [34], [35], there are very few works for describing the extraction process using multi-frequency ultrasound, as well as rarely exploring to optimize the extraction variables via uniform design approach.

For those purposes, in the present work, bioactive compounds from purple eggplant peels were extracted using single-, dual-, and tri-frequency ultrasound. The effects of different extraction variables on the extraction process were evaluated. Uniform design combined with partial least-squares (PLS) regression analysis was firstly employed to evaluate and optimize the different factors affecting the extraction. Based on the optimization results, The extraction yield of TPC and TAM assisted with ultrasound in different power levels and frequencies were further investigated.

## 2 Materials and methods

### 2.1 Materials and reagents

Purple eggplant was purchased from a food market in Yuanzhou district, Yichun city (China). The peels were removed from the eggplant using a sharp knife, and then dried in a vacuum oven at 30 °C (to avoid phenolic compounds damage) till a constant weight. The dried peels were pulverized into powder and sieved to different sizes (mean diameter) of 0.096, 0.226, 0.325, 0.815 and 0.998 mm. All powder samples were packed into different plastic bags and stored at 4 °C in a refrigerator prior to use.

All reagents used in this work including absolute ethanol, methanol, acetone, gallic acid, acetic acid, sodium carbonate, potassium chloride, hydrochloric acid, and Folin–Ciocalteu reagent were analytical grade and obtained from the Tianjin Chemical Factory (Tianjin, China).

### 2.2 Multi-frequency ultrasound extraction of bioactive compounds

The dried powders of purple eggplant were extracted with the used solvent in a multi-frequency ultrasonic cleaning instrument (Ningbo Xinzhi Biotechnology Co., ltd., Ningbo, China), which was composed of four plate ultrasonic generators with frequencies of 25, 40, 70 and 90 kHz. The nominal output power of each ultrasound generator can be adjusted from 0 to 300 W. The actual power dissipated in the bath was determined by the calorimetric method [36]. The ultrasound energy density was then calculated by dividing the measured power by the volume of solution inside. The entire system can operate in one of two modes: dual-frequency ultrasound with any combination of two different single-frequencies (X + Y), and tri-frequency ultrasound in random combination of three different single-frequencies (X + Y + Z), where X, Y and Z represent one of the frequencies of 25, 40, 70 and 90 kHz respectively.

### 2.3 Experimental design

#### 2.3.1 Single-factor experiment

Single-factor experiments for the extraction of bioactive compounds from purple eggplant peels were performed by varying solvent type (ethanol, methanol, acetone, water), ethanol concentrations (20 %, 35 %, 50 %, 65 %, 80 %), extraction temperatures (30, 35, 40, 45, 50 °C), particle size (0.096, 0.226, 0.325, 0.815, 0.998 mm), solid–liquid ratios (1:10, 1:20, 1:30, 1:40, 1:60 g/mL) and extraction times (10, 20, 30, 40, 50 min).

#### 2.3.2 Uniform design (UD)

Based on the results of the single-factor experiment, the rough range of each single factor was determined. uniform design, which can easily evaluate the effects of various single factors on responses, then was used to determine the optimum extraction conditions by combined with partial least-squares (PLS) regression analysis [37]. The regression equation model of the factors and the responses is expressed as follow:

$$Y_{k}=\alpha_{0}+\sum_{i=1}^{n}\alpha_{i}X_{i}+\sum_{i=1}^{n}\alpha_{\mathrm{ii}}X_{i}X_{i}+\sum_{i<j}^{n}\alpha_{\mathrm{ij}}X_{i}X_{j}$$

Where $Y_{k}$ are the predicted responses,$X_{i}$ and $X_{j}$ are the independent factors,$\alpha_{\mathrm{ij}}$ are the quadratic coefficients, $\alpha_{i}$ are the linear coefficients, and $\alpha_{0}$ is the constant coefficient.

The software named as Data Processing System (DPS, version 9.50) was applied to analyze the results and produce the regression model. When the experimental data were analyzed by DPS software, the standard regression coefficients (SRC) of all factors or factor combinations were calculated, where the absolute values of SRC reflected the influence degree on the responses.

The extraction yields of TPC and TMA were taken as the two responses, and the extraction variables (*X*_1_: ethanol concentration, *X*_2_: particle size, *X*_3_: extraction temperature, *X*_4_: extraction time, and *X*_5_: solid–liquid ratio) were taken as independent factors. The six-level-five-factor uniform design was introduced to optimize the extraction conditions of TPC and TMA. The factor-level table for the trial of extraction process was shown in Table 1.

**Table 1:** Factor-level table for the trial of extraction process.

**factors**	**levels**					
	**1**	**2**	**3**	**4**	**5**	**6**
*X*_1_: ethanol concentration (%)	20	35	50	65		
*X*_2_: particle size (mm)	0.998	0.815	0.325	0.226		
*X*_3_: extraction temperature (°C)	30	35	40	45	50	60
*X*_4_: extraction time (min)	20	25	30	35	45	50
*X*_5_: solid–liquid ratio (g/mL)	1:10	1:20	1:30	1:40	1:60	1:70

#### 2.3.3 Impact of ultrasound power and multi-frequency ultrasound

According to the results optimized by UD, the effects of five different levels of ultrasonic powers of 100 W, 150 W, 200 W, 250 W and 300 W, respectively corresponding to the ultrasound energy density of 7.5, 15.3, 29.8 and 53.6 W/L, on the extraction of TPC and TMA were studied. Subsequently, the effects of multi-frequency ultrasound including single-frequency (25, 40, 70, 90 kHz), dual-frequency (25 + 40, 25 + 70, 25 + 90, 40 + 70, 40 + 90, 70 + 90 kHz) and tri-frequency (25 + 40 + 70, 25 + 40 + 90, 25 + 70 + 90, 40 + 70 + 90 kHz) on the extraction of TPC and TMA were also examined at the optimal ultrasound power and other optimal conditions.

### 2.4 Determination of total polyphenolic content

The total phenolic content (TPC) was determined by spectrophotometric method [38]. Folin-Ciocalteu reagent was used to measure the total phenols in purple eggplant peels. Standard curve for calculation of total phenolic content was plotted by using gallic acid (0–65 µg/mL). The phenolic content of the extract was expressed as milligrams of gallic acid equivalent per gram of purple eggplant peel powder (mg GAE/g).

### 2.5 Determination of total monomeric anthocyanins

Determination of total monomeric anthocyanins (TMA) was performed based on the pH-differential method [39] by using sodium acetate (0.4 M) and potassium chloride (0.025 M) buffer solutions adjusted to pH 4.5 and pH 1.5, respectively. Briefly, an aliquot of extract was separately mixed with the buffers and the absorbance was determined at 520 and 700 nm in a UV–vis spectrophotometer (Shanghai Analytical Instrument Overall Factory, China). The TMA content was determined in relation to cyanidin-3-glucoside (C3G) as milligrams of C3G equivalent per 100 g of purple eggplant peel powder (mg C3G 100/g) according to Eq. (1).

(1) $$\text{TMA (mg/g) =}\;\frac{\left[\left(A_{520}-A_{700}\right){\text{pH}}_{1.0}-\left(A_{520}-A_{700}\right){\text{pH}}_{4.5}\right]\times \text{MM}\times \text{DF}\times 10^{5}\times V}{\varepsilon \times \text{L}\times \text{m}}$$

where A_520_ and A_700_ are the absorbance at 520 and 700 nm, respectively, MM is the molar mass of C3G (449.2 g/mol), DF is the dilution factor, V is the extract volume (L), ɛ is the molar extinction coefficient of C3G (26,900 L/mol/cm), L is the bucket length (cm) and m is the powder weight of purple eggplant peel (g).

### 2.6 Statistical analysis

All the tests were performed in triplicate. For single-factor experiments, each data was average value of three parallel experiments. Discrepancies were analyzed using analysis of variance (ANOVA) with SPSS 20.0 (IBM Corp., USA). The significance of the difference was established at 95 % confidence level (p < 0.05). All results were expressed as the mean ± standard deviation (SD). Origin (version 9.1) was applied for the experimental design and data processing.

## 3 Results and discussion

### 3.1 Single factor results

**Fig. 1:**
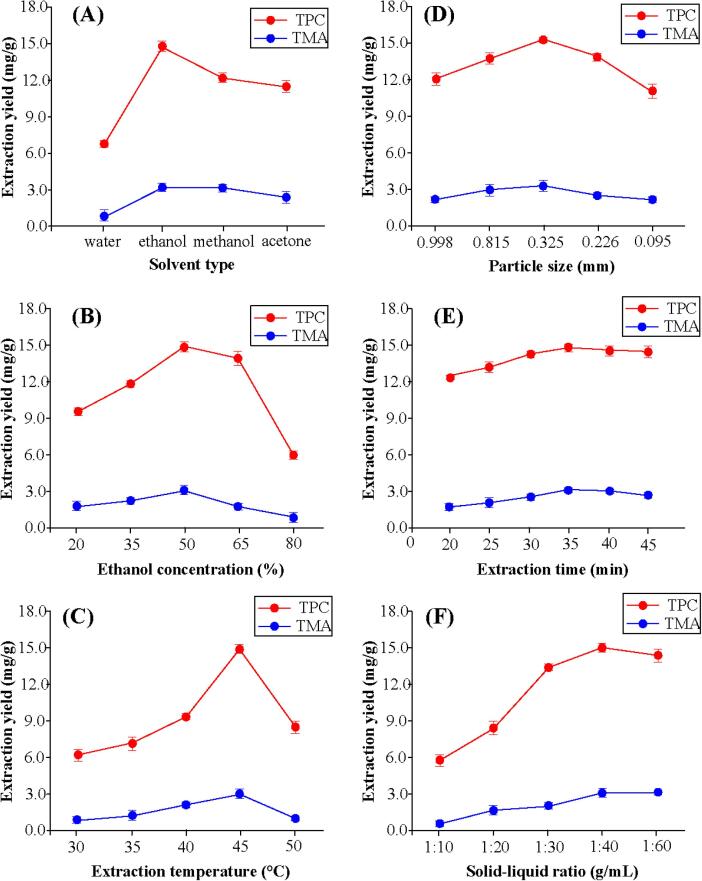
Effects of (A) solvent type, (B) ethanol concentration, (C) extraction temperature, (D) particle size, (E) extraction time and (F) Solid-liquid ratio on the extraction yield of bioactive compounds from purple eggplant peels.

As shown in Fig. 1, single factor experiments were performed for studying the influence of various variables on the extraction of TPC and TMA from eggplant peels.

#### 3.1.1 Effect of solvent type

Fig. 1**A** shows the effect of solvent type on the extraction yield of TPC and TMA at extraction temperature, particle size, extraction time and solid–liquid ratio of 45 °C, 0.325 mm, 35 min and 1:40 g/mL, respectively. It can be observed that four different solvents exhibited different effect on TPC and TMA under the same extraction condition. Ethanol is better for the extraction than other three solvents due to the highest values of TPC and TMA. This may be due to phenol and anthocyanins are all polar compounds with high polarities. So, other three solvents, with lower polarity than ethanol, exhibited lower extraction yield. This result is supported by previous works that reported ethanol gave the highest yield of phenolic compounds from different plant materials [40], [41]. So, ethanol was used as the extraction solvent in the subsequent experiments.

#### 3.1.2 Effect of ethanol concentration

According to above result, the effects of different ethanol concentrations on the extraction yield of TPC and TMA were further examined under the above extraction condition. Fig. 1**B** shows that both TPC and TMA values exhibited an increasing trend as ethanol concentration increased from 20 to 50 %. Butthe decreasing trends for them were observed as ethanol concentration further increased. Similar results have been reported that too low or too high ethanol solubility is not conducive to the extraction of oleanolic acid and ursolic acid from Ligustrum lucidum Ait [42] and of target cucurbitacin E from Iberis Amara seeds [43].

#### 3.1.3 Effect of extraction temperature

To further examine the effect of extraction temperature on the extraction of bioactive compounds from purple eggplant peels, other extraction conditions were fixed at particle size of 0.325 mm, extraction time of 40 min and solid–liquid ratio of 1:40 g/mL with ethanol concentration of 50 % as the extraction solvent. As illustrated in Fig. 1**C**, the mass transfer rate and vapor pressure of solute can be improved by increasing extraction temperature [44]. Thus, the extraction yield of TPC and TMA increased with the increase of temperature. However, when the temperature was above 45 °C, the extraction yields all showed a downward trend.This is consistent with the conclusion of the study on bioactive compounds extraction from propolis [45]. Itwas observed that high temperature has a negative influence on the extraction of bioactive compounds from purple eggplant peels although higher temperature increased the dispersion and solubility, which may be due to this reason that too high temperature is able to result in the degradation of thermosensitive substances [46].

#### 3.1.4 Effect of particle size

The effect of particle size on the extraction of bioactive compounds from purple eggplant peels have been studied when different particle size (0.998, 0.815, 0.325, 0.226 and 0.095 mm) were adopted under the extraction conditions as follows: ethanol concentration of 50 %, extraction temperature of 45 °C, extraction time of 40 min and solid–liquid ratio of 1:40 g/mL. As shown in Fig. 1**D**, the extraction yield of TPC and TMA were always increased gradually before the particle size of 0.325 mm, and then they reached the highest values. But a slight decrease in extraction yield of TPC and TMA was observed when particle sizes of 0.325–0.095 mm were adopted. This is attributed to the fact that too small particleremain at the surface, which leads to the reduction of extraction yield. Many similar results have been reported for the extraction of rutin from Sophora japonica [47], protein from sunflower meal [48] and curcuminoids from Curcuma longa [49].

#### 3.1.5 Effect of extraction time

For extraction time experiments, ethanol concentration of 50 %, extraction temperature of 45 °C, particle size of 0.325 mm and solid–liquid ratio of 1:40 g/mL were used as the extraction conditions. As indicated in Fig. 1**E**, the extraction yield of TPC and TMA showed the same change trend, which increased gradually before 35 min, then decreased slightly with the increase in extraction time. Liao et al. [50] also found that too long extraction time will reduce the extraction yield of flavonoids from peanut shells.This can be ascribed to the fact that phenols are easy to oxidized and hydrolyzed for a long time. Another possible reason is that the TPC and TMA from the inner section of particles of purple eggplant peels diffuse through the pores, which has a certain inhibitory effect on the extraction rate [51].

#### 3.1.6 Effect of solid–liquid ratio

The effects of different solid–liquid ratios were investigated under the follow extraction conditions: ethanol concentration of 50 %, extraction temperature of 45 °C, particle size of 0.325 mm and extraction time of 35 min. As shown in Fig. 3**F**, when the solid–liquid ratio increased from 1:10 to 1:40 g/mL, both TPC and TMA yields showed a prominent upward trend. The result is unsurprising because more solvent volume can lead to dissolve more constituents effectively [52]. But when it was higher than1:40 g/mL, TPC and TMA yield reached the final stabilization with the further increase in solid–liquid ratio, which due to the extracted solute becoming thinner in theliquid phase. Additionally, from the perspective of economy, the use of excessive solvents will be not conducive to the reduction in energy costand operating cost. Similar results have been reported for the extraction ofbaicalin and baicalein from Radix Scutellariae [53] and oleanolic and ursolic acids from Hedyotis diffusa [54].

### 3.2 Optimization experiments using uniform design

The major aim of this section is to determine the optimal extraction conditions for the TPC and TAM extracts. On the basis of the results of the single-factor experiments, The uniform design method was used to further refine the extraction process.

#### 3.2.1 Uniform design experimental findings

Based on the above results, five important factors (i.e., ethanol concentration, particle size, extraction temperature, extraction time and solid–liquid ratio) were investigated based on a U12 (4^2^ × 6^3^) uniform table and results of these experiments were shown in Table 2. The levels of these factors were presented in Table 1. A regression analysis was studied to fit the mathematical model according to the experimental data. By PLS regression analysis, regression equations of the two responses including TPC yield and TMA yield, which can describe the predicted model, were obtained as given in Eq. (2) and Eq. (3), respectively.

(2) $$\begin{array}{c} Y_{\text{TPC}}=\;10.756\;+\;4.3455X_{1}-\;3.4587X_{2}+\;1.1432X_{3}-\;3.1112X_{4}+\\ 2.0818X_{5}+\;0.9778X_{1}X_{1}+\;0.5553X_{2}X_{2}-\;0.8841X_{3}X_{3}-\;0.7498X_{4}X_{4}+\\ 0.9391X_{5}X_{5}-\;0.0933X_{1}X_{2}+\;0.0856X_{1}X_{3}+\;0.1006X_{1}X_{4}+\;0.0829X_{1}X_{5}\\ +0.0187X_{2}X_{3}+\;0.0095X_{2}X-\;0.0229X_{2}X_{5}+\;0.0137X_{3}X_{4}-\;0.0099X_{3}X_{5}+\\ 0.0074X_{4}X_{5}\ldots \left(R^{2}=0.986\right) \end{array}$$

(3) $$\begin{array}{c} Y_{\mathrm{TMA}}=\;2.7536\;-\;0.3652X_{1}+\;0.4302X_{2}+\;0.0985X_{3}-\;0.1547X_{4}+\\ 0.3365X_{5}+0.0532X_{1}X_{1}-\;0.0363X_{2}X_{2}+0.0621X_{3}X_{3}-\;0.0093X_{4}X_{4}+\\ 0.0412X_{5}X_{5}+\;0.0169X_{1}X_{2}-\;0.0905X_{1}X_{3}+\;0.0556X_{1}X_{4}-\;0.0813X_{1}X_{5}+\\ 0.0034X_{2}X_{3}-\;0.0155X_{2}X+\;0.0029X_{2}X_{5}-\;0.0035X_{3}X_{4}+\;0.0057X_{3}X_{5}+\\ 0.0013X_{4}X_{5}\ldots \left(R^{2}=0.975\right) \end{array}$$

**Table 2:** U12 (4^2^ × 6^3^) uniform design with the observed responses and experiment results.

**test times**	**factors**					**responses**	
	**ethanol concentration (%)**	**particle size (mm)**	**extraction temperature (°C)**	**extraction time (min)**	**solid**–**liquid ratio (g/mL)**	***Y*_TPC_** **(mg/g)**	***Y*_TMA_** **(mg/g)**
R1	2	4	1	3	4	15.15 ± 1.02	3.56 ± 0.05
R2	4	3	3	6	2	15.02 ± 0.69	2.71 ± 0.23
R3	3	2	2	6	5	15.03 ± 1.47	3.01 ± 0.15
R4	4	1	4	2	6	14.99 ± 0.08	2.98 ± 0.66
R5	1	1	2	4	3	14.87 ± 1.23	3.45 ± 0.65
R6	2	3	5	1	5	15.46 ± 2.14	2.96 ± 0.78
R7	3	1	6	4	4	14.98 ± 1.82	3.23 ± 0.07
R8	1	4	4	5	6	14.82 ± 0.78	2.72 ± 0.85
R9	2	2	6	5	1	15.18 ± 1.26	3.06 ± 0.03
R10	4	4	5	3	3	14.80 ± 0.79	2.81 ± 0.05
R11	3	3	1	2	1	14.77 ± 1.31	3.09 ± 0.18
R12	1	2	3	1	2	15.42 ± 0.93	2.76 ± 0.03

#### 3.2.2 Interaction effects between various factors

**Fig. 2:**
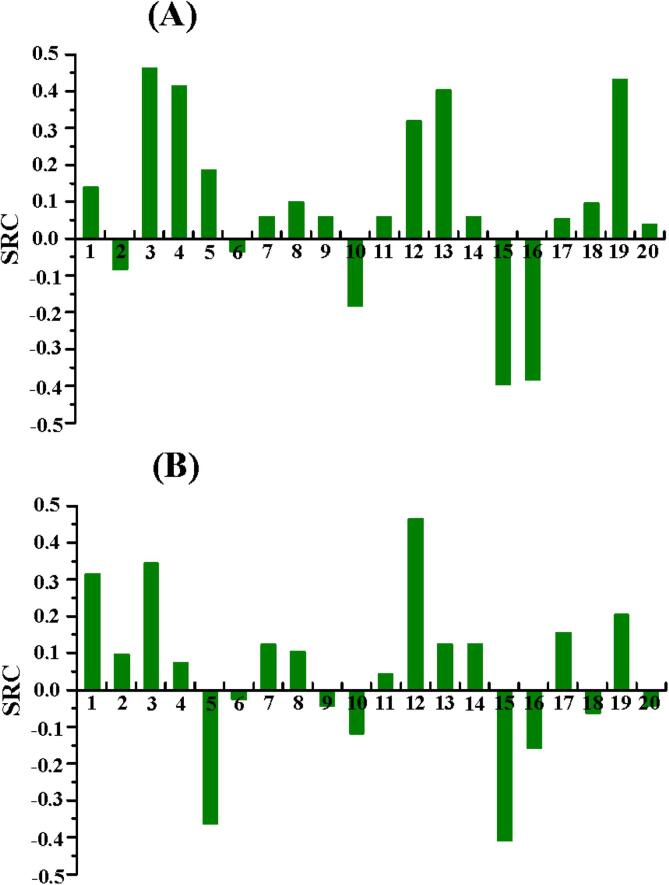
The SRC values of each factor or factor combination when the responses were TPC yield (A) and TMA yield (B) from purple eggplant peels.

The SRC values of five factors (1, 2, 3, 4 and 5 represented linear coefficients for *X*_1_, *X*_2_, *X*_3_, *X*_4_ and *X*_5_, respectively) or fifteen factor combinations (6, 7, 8, 9 and 10 represented quadratic coefficients for *X*_1_^2^, *X*_2_^2^, *X*_3_^2^, *X*_4_^2^ and *X*_5_^2^, respectively; 11, 12, 13, 14, 15, 16, 17, 18, 19 and 20 represented *X*_1_*X*_2_, *X*_1_*X*_3_, *X*_1_*X*_4_, *X*_1_*X*_5_, *X*_2_*X*_3_, *X*_2_*X*_4_, *X*_2_*X*_5_, *X*_3_*X*_4_, *X*_3_*X*_5_ and *X*_4_*X*_5_, respectively) for the two responses were shown in Fig. 2, where A and B corresponded to the responses of TPC and TMA, respectively.

As shown in Fig. 2A, the extraction temperature (3 in Fig. 2A) and extraction time (4 in Fig. 2A) were the two independent factors that significant effects on TPC yield. The ethanol concentration (*X*_1_) and particle size (*X*_2_) affected the TPC yield in the form of the combination of *X*_1_*X*_3_ (12 in Fig. 2A), *X*_1_*X*_4_ (13 in Fig. 2A), *X*_2_*X*_3_ (15 in Fig. 2A) and *X*_2_*X*_4_ (16 in Fig. 2A) rather than in the form of a single factor. Additionally, the great interaction effects of extraction temperature (*X*_3_) and solid–liquid ratio (*X*_5_) (19 in Fig. 2A) was also obviously found, followed by the effect of independent solid–liquid ratio and its quadratic coefficient (10 in Fig. 2A).

The TMA yield was mainly influenced by the ethanol concentration (1 in Fig. 2B), the extraction temperature (3 in Fig. 2B), the solid–liquid ratio (5 in Fig. 2B), and the factor combinations which contained extraction temperature such as *X*_1_*X*_3_ (12 in Fig. 2B) and *X*_2_*X*_3_ (15 in Fig. 2B). The effect of the combination of the ethanol concentration and the extraction temperature (12 in Fig. 2B) on TMA yield was remarkable (|SRC|=0.47), whereas the effects of the independent extraction time, particle size on the extraction of TMA (2 and 4 in Fig. 2B) were very limited (|SRC| < 0.08).

#### 3.2.3 Optimization results by DPS and validation

According to PLS regression analysis combined with DPS software, the optimum extraction conditions for the extraction of bioactive compounds from purple eggplant peels were shown in Table 3. For TPC, the ideal extraction conditions were: ethanol concentration of 53.6 %, extraction temperature of 44.5 °C, particle size of 0.336 mm, extraction time of 35.2 min and solid–liquid ratio of 1:43 g/mL. For TMA, the optimal extraction conditions were: ethanol concentration of 52.5 %, extraction temperature of 45.8 °C, particle size of 0.297 mm, extraction time of 35.6 min and solid–liquid ratio of 1:42 g/mL.

**Table 3:** Optimization results for the extraction of bioactive compounds from purple eggplant peels.

	**ethanol concentration (%)**	**particle size (mm)**	**extraction temperature (°C)**	**extraction time (min)**	**solid**–**liquid ratio (g/mL)**
TPC	53.6 %	0.336	44.5	35.2	1:43
TMA	52.5 %	0.297	45.8	35.6	1:42

Under the optimal extraction conditions, the experimental values were compared with the predicted values obtained by the data processing system. As shown in Table 4, the relative errors for TPC and TMA were 3.26 % and 4.59 % respectively, indicating the experimental results were consistent with the model. Thus, the model developed (as shown in Eq. (2) and Eq. (3)) can accurately predict the extraction yield of bioactive compounds from purple eggplant peels.

**Table 4:** Comparison of predicted value with experimental results.

	**predicted value**	**experimental value**	**relative error (%)**
Yield of TPC (mg/g)	15.12	15.61 ± 1.02	3.26
Yield of TMA (mg/g)	3.23	3.38 ± 0.06	4.59

### 3.3 Impact of multi-frequency ultrasound on the extracton of TPC and TMA

Many studies have proved that ultrasound can effectively improve the extraction rate and sonochemical yield. For instance, phenolics compounds of the eggplant peels extracts after assisting by ultrasound increased by 25.58 % compared with the conventional grinding extraction method [55]. It was found that there were significant differences in the efficiency of biological extraction with different ultrasonic frequencies and power, which mainly comes from two aspects [56], [57], [58], [59]. On the one hand, the extraction efficiency or the concentration of products in the extraction solution is highly related to the ultrasonic frequency and power. On the other hand, the biological substances and cell types in the extracted objects are also highly correlated with the ultrasonic frequency and power. Different biological substances and cells have different sensitivities to different frequencies of ultrasonic waves. Therefore, specific optimization of ultrasonic frequency and power tests need to be designed.

#### 3.3.1 Influence t of ultrasound power

**Fig. 3:**
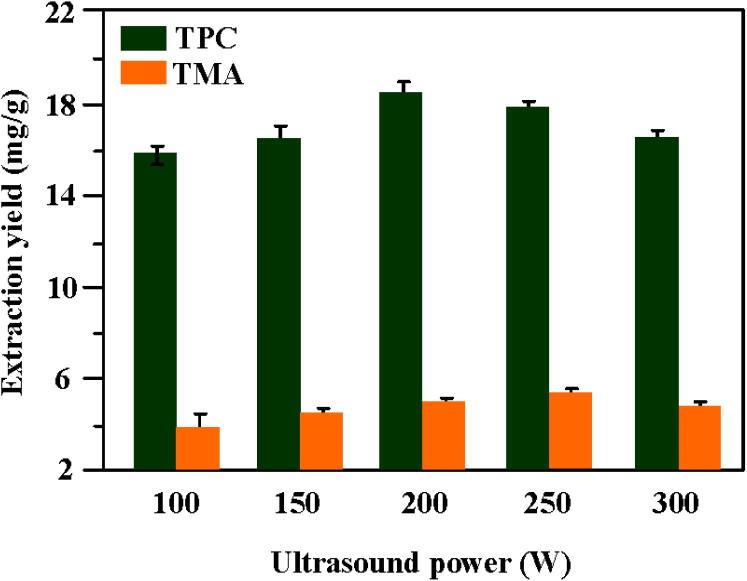
The influence of ultrasound power on the extraction of TPC and TMA at 40 kHz and under other optimal conditions.

The influence of ultrasound power on the extraction of bioactive compounds from purple eggplant peels had been performed when different ultrasound powers (100 W, 150 W, 200 W, 250 W and 300 W) were adopted at extraction temperature of 44.5 °C, particle size of 0.336 mm, extraction time of 35.2 min, ultrasonic frequency of 40 kHz and solid–liquid ratio of 1:43 g/mL with 53.6 % ethanol concentration as the extraction solvent. As shown in Fig. 3, when ultrasound power increased, both TPC and TMA exhibited a similar trend, which always increased before 200 W and then reached the highest value. After that, both extraction yields slightly decreased as the ultrasonic power was further increased. Similar results were reported in other research for MUAE of anthocyanin from strawberry fruit [60], and UAE of total anthocyanins from purple sweet potatoes [61]. This may be due to the mechanical effect, thermal effect, and cavitation effect are all strengthened with the increase of ultrasonic power. As a result, the ultrasonic cavitation intensity is increased. Thus, more extracts are rapidly released from plant cells. But when ultrasound power is too low or too high, the cavitation intensity is very weak or will not even occur [62], [63].

#### 3.3.2 Influence t of ultrasound frequency

**Fig. 4:**
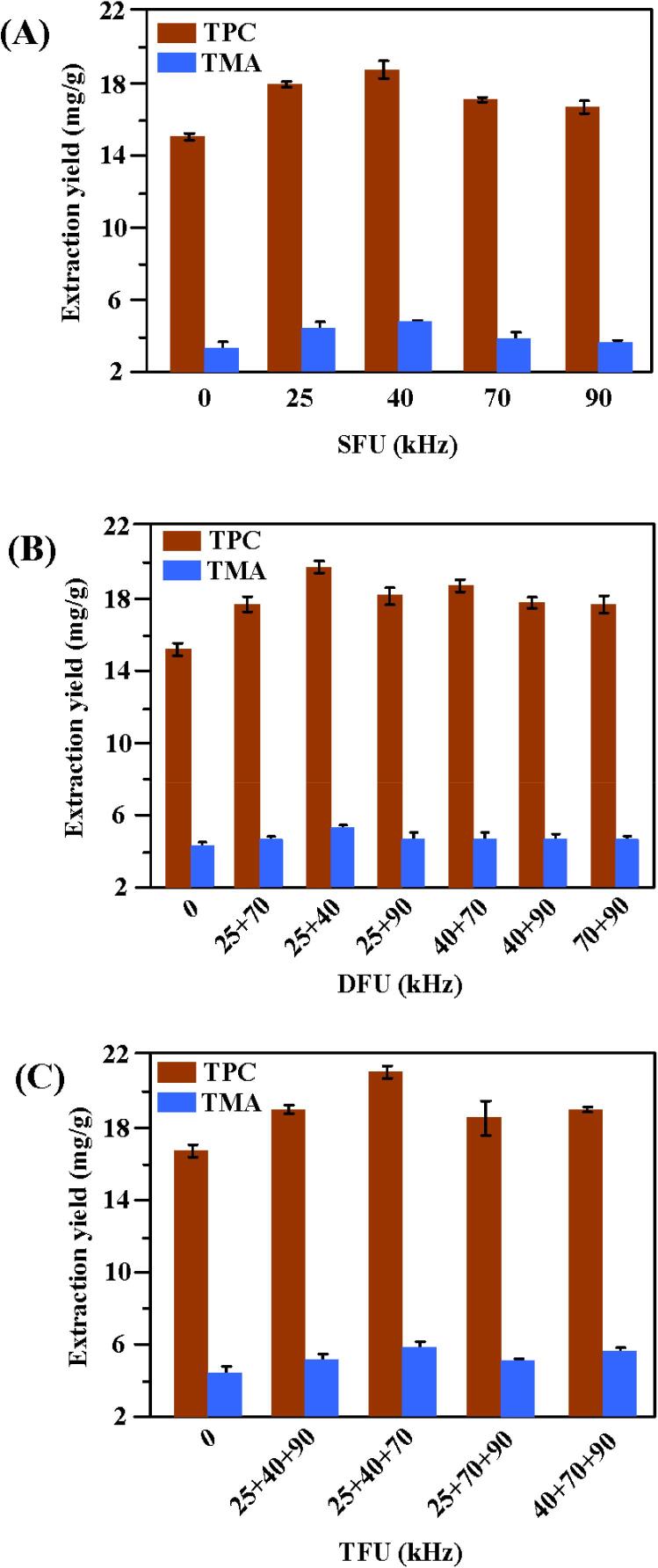
The influences of multi-frequency ultrasound (A: Single-frequency ultrasound (SFU); B: Dual-frequency ultrasound (DFU); C: Tri-frequency ultrasound (TFU)) on the extraction of bioactive compounds from purple eggplant peels under other optimal conditions.

According to the above optimization experiment of ultrasound power, the influence of different single-frequency, daul-frequency and tri-frequency ultrasound on the extraction of TPC and TMA were shown in Fig. 4. It can be seen that ultrasonic frequency has a few effects on the extraction of TPC and TMA when other conditions are the same.

The results (Fig. 4**A**) showed that The yield of TPC and TMA extracted by different single-frequency were in the following sequence from high to low: 40 kHz > 25 kHz > 70 kHz > 90 kHz > 80 kHz > non-ultrasound. Under the single-frequency extraction of 40 kHz, the yield of TPC and TMA reached the highest value, which was higher than that of other single-frequencies or no ultrasound treatment. Fig. 4**B** showed the effects of different daul-frequency ultrasound in simultaneous modes on the extraction yield of TPC and TMA. It can be observed that the daul-frequency of 25 + 40 kHz gave the highest yield for both TPC and TMA. Overall, compared to the single-frequency ultrasound, the yields of TPC and TMA of daul-frequency ultrasound were higher. This finding is consistent with the previous research on the extraction of total phenolic and flavonoid contents from hardy kiwi (*Actinidia arguta* L.) leaves using multi-frequency ultrasound [64]. It can be seen from Fig. 4**C** that tri-frequency ultrasound in simultaneous mode also has significant effect on the extraction of TPC and TMA. Among them, 25 + 40 + 70 kHz tri-frequency in simultaneous mode the maximum output to TPC and TMA extraction compared with other tri-frequencies and on ultrasound. It also can be clearly found that the yield of TPC and TMA by different frequencies ultrasound treatment were in the following sequence: 25 + 40 + 70 kHz > 25 + 40 kHz > 40 kHz. Under the tri-frequency ultrasound of 25 + 40 + 70 kHz, both TPC and TMA yields achieved the highest values (i.e., 20.37 mg/g for TPC and 5.22 mg/g for TMA), which were 18.76 % and 23.65 % higher than that of 25 + 40 kHz and 40 kHz, respectively.

The possible main reasons for these phenomenons are that ultrasound is a kind of mechanical wave, which can produce severe cavitation effect in liquid medium. Tri-frequency ultrasound can generate greater mechanical force and more cavitations because it can effectively eliminate the standing wave generated by single-frequency ultrasound. Thus, tri-frequency ultrasound can produce more cavitation bubbles to collapse than that of single-frequency ultrasound, which lead to a higher temperature and pressure, therefore cause a greater disruption for blueberry pomace cells [65]. Under daul-frequency ultrasound, the cavitation bubbles cannot collapse in time since the bubbles movement frequency is changing randomly. As a result, the strong cavitation effects become difficult to take place, which was the possible reason that the extraction under dual-frequency ultrasound showed a low TPC and TMA yield, while the extraction showed a higher yield owing to the improved temperature and molecule movement under tri-frequency ultrasound [66], [67].

### 3.4 Comparison of multi-ultrasound and conventional reflux extraction

In order to compare the extraction efficiency and make the comparison, the conventional reflux extraction (CRE) method was carried out at different extraction conditions with different levels, which were optimized as follows: ethanol concentration of 70 %, temperature of 60 °C, extraction time of 80 min. Other optimized conditions were the same as the optimal UAE including single-, dual-, and tri-frequency ultrasound. The obtained results (Table 5) showed significantly lower values for TPC (9.75 ± 0.11 mg/g) and TMA (2.01 ± 0.09 mg/g) in CRE method. Comparison with CRE, the use of ultrasonic irradiation, especially tri-frequency ultrasound had achieved the highest yield of TPC (21.07 ± 0.87 mg/g) and TMA (5.88 ± 0.39 mg/g), which were 2.16 times and 2.94 times that of CRE. In addition, it is worth noting that the ethanol concentration, temperature and extraction time used by ultrasound extraction were lower than that of CRE ones, which indicated that UAE is a method of more energy-saving, time-saving and higher extraction efficiency. Moreover, due to the coupling effect between different ultrasonic frequencies, tri-frequency ultrasound can obtain the highest extraction yield of TPC and TMA.

**Table 5:** Comparison of the results between MFU, DFU, TFU and CRE methods.

**Methods**	**Ethanol concentration (%)**	**Particle size (mm)**	**Temperature (°C)**	**Time (min)**	**Solid-liquid ratio (g/mL)**	**Frequency (kHz)**	**Power (W)**	**Yields (mg/g)**	
								**TPC**	**TMA**
MFU	50	0.325	45	35	1:40	40	200	18.75 ± 1.06	4.58 ± 0.64
DFU	50	0.325	45	35	1:40	25 + 40	200	19.69 ± 0.93	5.04 ± 0.28
TFU	50	0.325	45	35	1:40	25 + 40 + 70	200	21.07 ± 0.87	5.88 ± 0.39
CRE	70	0.325	60	80	1:40	—	—	9.75 ± 0.11	2.01 ± 0.09

## 4 Conclusions

The results from this work showed that the extraction temperature and extraction time were significant effects on TPC yield. The ethanol concentration and particle size affected the TPC yield in the form of the combination. Also, the interaction effects between extraction temperature and solid-liquid ratio was great, followed by independent solid-liquid ratio and its quadratic coefficient. For TMA, its yield was mainly influenced by the ethanol concentration, extraction temperature, solid-liquid ratio, and the factor combinations that contained extraction temperature. The effect of the combination of the ethanol concentration and extraction temperature was remarkable, whereas the independent extraction time, particle size on the extraction of TMA were very limited.

Additionally, it was found that multi-frequency ultrasound is an efficient approach to improve the extraction of bioactive compounds from purple eggplant peels. It also can be found that the yield of TPC and TMA by different frequencies ultrasound treatment were in the following sequence: 25 + 40 + 70 kHz > 25 + 40 kHz > 40 kHz. Under 25 + 40 + 70 kHz, both TPC and TMA yields reached the highest values (20.37 mg/g and 5.22 mg/g). So, it is hoped that tri-frequency ultrasound can play an increasingly important role in the extraction of bioactive components from natural substances in the future.

## CRediT authorship contribution statement

**Jianqing Liao:** Project administration, Formal analysis, Validation, Resources, Methodology, Software, Writing – original draft, Writing – review & editing, Conceptualization, Supervision, Investigation, Data curation, Visualization, Funding acquisition. **Hongkun Xue:** Investigation, Methodology, Validation, Data curation, Formal analysis, Project administration, Writing – review & editing, Funding acquisition. **Junling Li:** Investigation, Software, Methodology, Formal analysis, Writing – original draft.

## Declaration of Competing Interest

The authors declare that they have no known competing financial interests or personal relationships that could have appeared to influence the work reported in this paper.

## Data Availability

The data that has been used is confidential.
